# Probiotic based-diet effect on the immune response and induced stress in irradiated mass reared *Ceratitis capitata* males (Diptera: Tephritidae) destined for the release in the sterile insect technique programs

**DOI:** 10.1371/journal.pone.0257097

**Published:** 2021-09-10

**Authors:** Meriem Msaad Guerfali, Kamel Charaabi, Haytham Hamden, Wafa Djobbi, Salma Fadhl, Amor Mosbah, Ameur Cherif

**Affiliations:** 1 Laboratory of Biotechnology and Nuclear Technologies LR16CNSTN01, National Center of Nuclear Sciences and Technologies, Ariana, Tunisia; 2 Laboratory of Biology and Bio-Geo Resources LR11ES31, Higher Institute of Biotechnology of Sidi Thabet, University of Manouba, Ariana, Tunisia; University of Carthage, TUNISIA

## Abstract

*Ceratitis capitata* (medfly) is one of the most devastating crop pests worldwide. The Sterile Insect Technique (SIT) is a control method that is based on the mass rearing of males, their sterilization, and release in the field. However, the effectiveness of the technique depends on the quality of the released males and their fitness. We previously isolated and selected a probiotic bacteria (*Enterobacter* sp.), from wild-caught medflies, according to criteria that improved biological quality traits of reared medfly males.We firstly evaluated the impact of the irradiation on the expression of different immune and stress genes in the medfly sterile males. Expression was measured at differents time points ranging from 0 to 168 h after irradiation to capture the response of genes with distinct temporal expression patterns. Then, we supplemented the larval diet with previously isolated *Enterobacter* sp.strain, live and autoclaved at various concentrations to see whether the probiotic treatments affect, through their protective role, the gene expression level, and quality traits. The irradiation had significant effect on the genes *attacin*, *cecropin*, *PGPR-LC*, *hsp23*, and *hsp70* level expression. The expression of *attacin* and *PGPR-LC* was up-regulated while that of *cecropin* was down-regulated. *Hsp* genes showed decreased levels between 0 and 18 h to peak at 72 h. However, the supplementation of the probiotic strain, either live or autoclaved, was statistically significant only for *attacin*gene. However, significant interaction time x probiotic was noticed for *attacin*, *cecropin*, *hsp23* and *hsp70*. The probiotic treatments also improved the quality control parameters like pupal weight. From this work we can conclude that a consortium of parabiotics (autoclaved probiotics) treatment will be recommended in insectaries considering both the beneficial effects on mass reared insects and its general safety for insectary workers and for environment.

## 1. Introduction

*Ceratitis capitata* Wideman (Diptera: Tephritidae) is considered a major fruit fly pest of economic importance attacking more than 300 different hosts [1, 2]. The level of the economic damage of medfly, as it is commonly called, is very high because of its polyphagia [3]. Conventional chemical pesticides are applied to target the medfly adult stage [4]. Nevertheless, it is worth noting that they have unfavorable environmental effects and serious health consequences for farm operators [5, 6] and consumers [7, 8]. This implies the need for developing novel effective and environmentally sound pest management approaches as sustainable alternatives to chemical control.

Sterile Insect Technique (SIT) has shown evidence and effectiveness in area-wide integrated pest management programs (AW-IPM) against medfly [9]. SIT is based on the release of overflooding ratios of sterilized medfly males to target wild populations [10, 11]. However, SIT efficiency depends on the performance of the produced and released males. Therefore, the performance of the males is a prerequisite of success for the technique [12, 13]. These males have to achieve competitiveness in a way to switch the mating behavior of wild females to refractoriness [14]. However, genetic sexing of the laboratory strain, colonization, mass-rearing conditions, and irradiation impact negatively on the performance of sterile medfly males [15–24].

Overall, there is convincing evidence that the structure of intestinal microbiota can play an important role in the performance of sterile insects. The gut medfly symbiotic community is comprised of predominant bacterial genera such as *Klebtiella*, *Enterobacter*, *Citrobacter*, *Morganella*, *Providentia*, and *Pantoae* [25–30]. More recently, Malacrino et al. [31] have reported different community composition belonging to the phylum of Proteobacteria, including genera of (i) Alphaproteobacteria such as Acetobacter and Gluconobacter; (ii) Betaprotobacteria such as Burkholderia and (iii) Gammaproteobacteria such as Pseudomonas. Additionally, small Firmicutes and minor Actinobacteria were also detected by Nikolouli et al. [32].

Rearing stressors could impair this composition and this has been demonstrated for medfly [26–29, 33]. This dysbiosis gives an advantage to bacterialgenera such as *Providencia* and *Pseudomonas* considered as potential pathogens for the fly. Interestingly, it was shown that the supplementation of the larval diet with probiotics could repair an unbalanced gut microbiome and improve the fitness of the flies, counteracting the effects due to mass rearing stressors. Enrichment with probiotics,such as *Enterobacter* sp., of the medfly larval diet [28, 29] and *Klebtiella oxytoca* for the adult diet [34] has been recommended in insectaries.

Besides assessing quality control parameters that reflect mass-reared insects’ fitness, endogenous gene regulationto invaders and environmental stressors should be evaluated.

Stress responses and immune responses are different, although intimately linked. Stress genes are induced and modulated within insects. These stress resistance genes can play a protective role and represent stress indicators such as antioxidant proteins (e.g, superoxyde dismutase, catalase, glutathione peroxidase andthioredoxin) [35] and heat shock proteins (hsps, hsp23s, hsp70s, hsps90). Heat shock proteins (Hsps) are playing a crucial adaptive role in stress tolerance within insects [36]. They are molecular chaperones that are coded by a subset of a larger group of genes. The functions of Hsps include transport, folding, unfolding, assembly/disassembly, and degradation of misfolded or aggregated proteins [37, 38]. Their regulation is a common cellular response for each insect species. It is a balance between benefits and costs (negative impact on growth, development rate, and fertility). For example, high levels of hsp70 were reported to decrease or even retard growth and cell division [39, 40], and may also reduce reproduction [41, 42]. Moreover, irradiation has been reported to induce high levels of *hsp70* gene in some insects via the production of ROS (reactive oxygen species) in cells [43–48].

On the other hand, systemic immune response, manifested by the secretion of antimicrobial peptides (AMPs) from cells of the fat body into the hemolymph (e.g., cecropin, defensin, relish, PGRP, andattacin),is induced in response to bacterial infection or injury [49]. The regulation of the insects’ anti-microbial peptides (AMPs) is modulated through the Imd and Toll signaling pathways [50–52]. It seems that the native insectgut microbiota is stimulating the secretion of AMPs against pathogens while achieving immune tolerance to the commensal gut microbial community [53]. RNAi silencing experiments on *Drosophila* sp. flies demonstrated that alteration of the gut microbiota is accompanied by increased mortality [54].

Interestingly, Valanne et al. [55] demonstrated that infectious agents and irradiation induced the same canonical pathways within insects. Irradiation of *Drosophila* sp. at low doses generated the transcription of AMPs of the Toll pathways such as cecropin, defensin, and metchnikowin [56].

The aim of this work was to provide a first insight into how irradiation and probiotic treatments could affect mass reared *C*. *capitata* males immunity and gain a better understanding of the interaction of these two factors on insect physiology. To do this, we studied the expression of *attacin*, *cecropin*, *PGPR-LC*, *hsp23* and *hsp70* (as representative genes of immune and stress response) on medfly males exposed to irradiation and after enrichment with live and autoclaved probiotic. The immune and stress genes *attacin*, *cecropin*, *PGPR-LC*, *hsp23* and *hsp70* were selected to obtain a general view of the immune activity. Expression was measured at different time points ranging from 0 to 168 h after irradiation to capture the response of genes with distinct temporal expression patterns. The medfly sterile male quality control parameters such as pupal weight, emergence and flight ability were also assessed.

## 2. Materials and methods

### 2.1. Fly stock

The Mediterranean fruit flies were obtained from a stock colony of the VIENNA 8 genetic sexing strain (GSS) maintained at the laboratory of sterile insects at the National Centre of Nuclear Sciences and Technologies of Tunisia (CNSTN). This strain is known to carry a temperature-sensitive lethal mutation (*tsl*), which makes the females sensitive to temperatures above 32°C. The females carry a second mutation resulting in white pupae (*wp*), unlike the male pupae which are brown [57]. Adult flies were kept in cages with two sides covered with a mesh to allow oviposition. The cages were provided with water and yeast hydrolyzate (3:1 ratio). Eggs were collected daily in water containers placed below a mesh cover. The third instar larvae leave the larval medium to trays with sawdust for pupation [58].

### 2.2. Experimental design

To investigate the effect of the irradiation on the immune response, male pupae were irradiated two-days before emergence (0 h). The molecular analyses were carried out on individuals at irradiation time (0 h) (pupal stage, two-days before emergence) after 18 hours (pupal stage, 30 hours before emergence), at 72 hours (one-day-old adult) and at 168 hours (five-days-old adult) of irradiation. Other pupae from the same production batch were withheld from irradiation and served as non-irradiated, control males.

**Fig 1:**
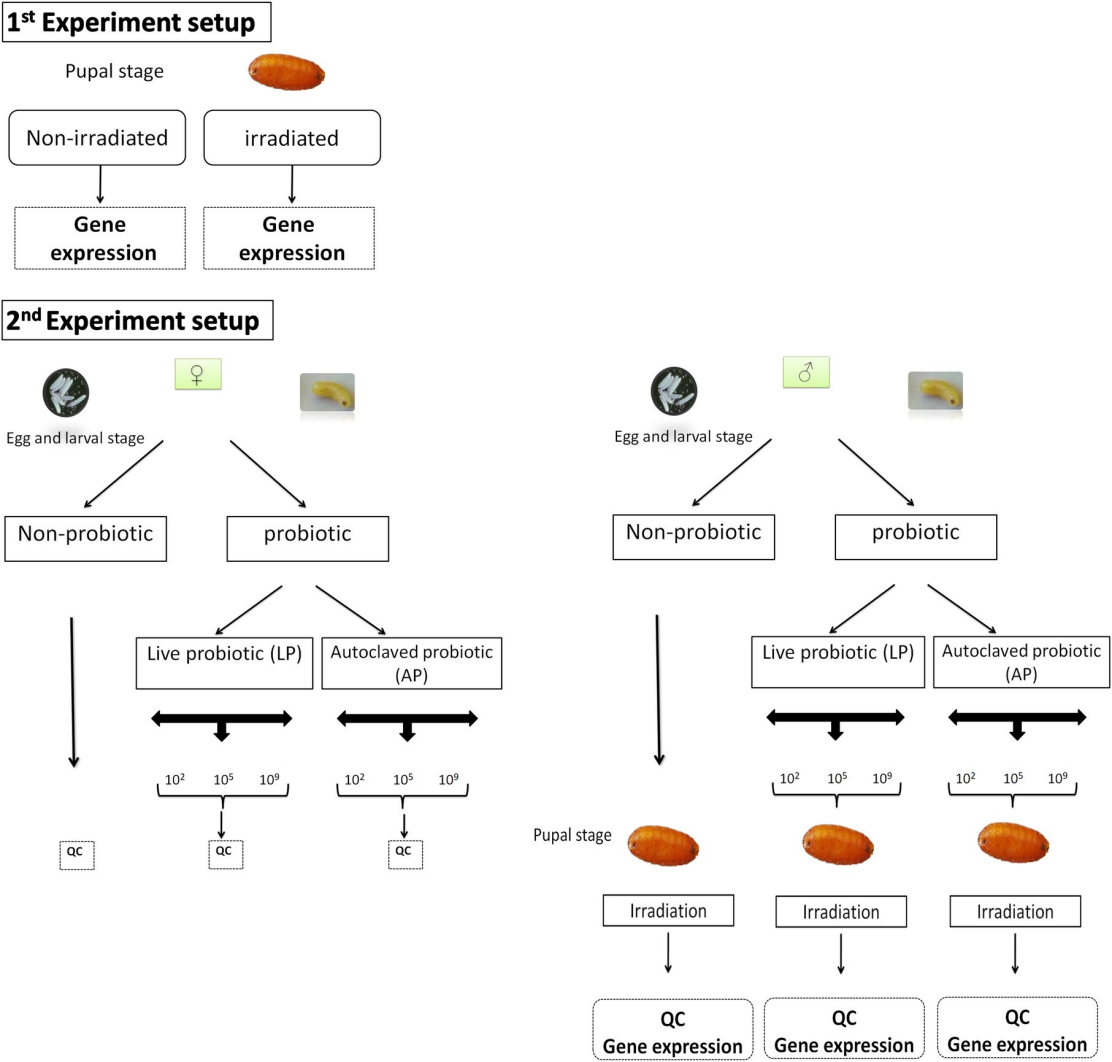
Summary of the experimental design.

To investigate the effect of the probiotic-based diet on medfly males and females, we enriched the larval diet with the selected probiotic at three concentrations (10^9^, 10^5^ and 10^2^ CFU/ml). To distinguish between bacteria having an effect either through interaction with the insects or just as nutrient source, both autoclaved (AP) and live (LP) bacteria of the same concentration were used. Control group was fed the artificial diet without probiotic (Non-probiotic). The molecular analyses were carried out on medfly males (AP and LP) at the same time points, 0 h, 18 h, 72 h and 168 h after irradiation (Fig 1).

Irradiated males and non-irradiated females were subjected to quality control parameters tests to assess the effect of probiotic.

### 2.3. Larval diet preparation

The probiotic strain *Enterobacter* sp. (KY810513) was isolated from wild-caught Tunisian medflies [59]. The strain was selected through *in vitro* and *in vivo* probiotic criteria such as biofilm formation, tolerance to irradiation, exopolysaccharides (EPS) production, and quality control parameters enhancement. The bacterium was grown in sterile Luria-Bertani (LB) broth up to the mid-log phase, then quantified on LB agar medium. Aliquots of 10^2^, 10^5^, and 10^9^UFC/ml of live (LP) and autoclaved (AP) bacterial suspensions per gram of larval diet were added to 100 g [28]. Two hundred eggs were surface-sterilized with quaternary ammonium at 150 ppm for 1 minute [21] and seeded on 100g of each treated larval diet. Eggs were not subjected to thermal treatment, males and females choice was based on the color of the pupae (*wp* mutation) and the difference in the development time between both sexes. Four replicates were performed for each bacterial suspension type and concentration.

### 2.4. Irradiation procedure

Two days before emergence male pupae were irradiated in a Cobalt-60 irradiator designed for foodstuff and sterilization of medical equipment. An irradiation device was installed, designed specifically for the irradiation of *C*. *capitata* pupae, consisting of 4 turntables that make it possible to rotate the canisters holding the pupae within the radiation field [22, 58]. The axis of rotation is vertical and parallel to the source pencils. Male pupae were treated at 90 Gy with the dose-rate of 0.1KGy/h.

### 2.5. Pupal weight, adult emergence, and flight ability parameters

One hundred irradiated male pupae and non-irradiated female pupae from each treatment (LP and AP at three concentrations) were weighted. Treated pupae were placed in flight ability cylinders (10 cm high, 100 pupae per cylinder) for emergence [60]. The cylinders were placed in Plexiglas cages. Emerging flies were collected continuously. The test results were recorded 72 h after set up. After the adult emergence, the percentage of male and female fliers was evaluated. Three replicates were performed for pupae weight and ten for the emergence and flight ability test. Control flies for males were sterile pupae irradiated and fed on a control larval diet. For females, control flies were non-irradiated and fed on a control larval diet.

### 2.6. RNA isolation, DNAse treatment, and cDNA synthesis

Total RNA was extracted with Trizol from non-irradiated and irradiated medfly malesfrom each probiotic treatment or control condition at 0 h, 18 h, 72 h and 168 h after irradiation. Extraction of the RNA was followed by a DNase treatment (with DNase I (Thermo Fisher) according to manufacturer’s instructions) to eliminate potential genomic DNA in the samples. RNA was then stored at -80°C before further processing. The quality and quantity of RNA were assessed with a Nanodrop ND-1000 spectrophotometer (Nanodrop Technologies). We only considered samples with a 260/280 ratio superior to 1.8 and a 260/230 ratio superior to 2.0 [61]. cDNA was produced using SuperScript™ VILO™ cDNA Synthesis Kit (Invitrogen, UK) in 20 μl total volume using 200 ng of total RNA, following the manufacturer’s protocol.

Non-irradiated pupae and adults were used as controlsfor the study of irradiation effect, and irradiated pupae and adults not treated with probiotics were used as controls for the study of probiotic effect (Fig 1) as indicated in the statistical analyses section.

### 2.7. Real-time quantitative PCR

The cDNA’s were used to assess the relative transcript abundance of the *cecropin1* (mentioned as *cecropin*), *attacin A* (mentioned as *attacin*), *PGPR-LC*, *hsp70*, and *hsp23-alpha* (mentioned as *hsp*23) genes. Two medfly reference genes (*GAPDH2* and *G6PDH*) were used for normalization [62]. Real-time PCR (RT-qPCR) was performed with the Super mix (Syber ® Premix Ex Taq TM (Tli RNaseH Plus, Takara). Cycling parameters were: 3 minutes at 95°C, 40 cycles of 10 seconds at 95°C and 30 seconds at the temperature of the respective primer pairs, and 30 seconds at 68°C. They were performed using the Bio-Rad DNA Engine Mini Opticon real-time PCR detector and SYBR green dye. A fluorescence reading was made at the end of each extension step. Melt-curve analyses were used after amplification to confirm that fluorescence was the result of amplified products of the predicted size. A 10-fold dilution series of cDNA was used to create the standard curve, and the qRT-PCR efficiency (E) values between 89% and 118% were determined for all primer pairs. The primers for *hsp70* and *hsp*23 were designed by Primer 3 program (http://simgene.com/Primer3 (Table 1). Primer pairs for *cecropin1*, *attacin* and, *PGRP-LC* are listed in Gomulski et al. [63]. Delta CT analyses were performed as described by Livak and Shmittgen [64].Values are presented as fold-differences relative to expression found in controls and as predicted values relative to the reference genes G6PDH and GAPDH2.

**Table 1:** Primers used for the qRT-PCR analyses.

Gene ID	Accession	Primer pair sequences	References
*G6PDH*	S67872	F: Cggacgagcaggcaaaatatg	Gomulski et al. (2012) [63]
		R:Agacggacggcggtaagg	
*GAPDH2*	FS831	F: Ggtcgcatcggtcgtctgg	Gomulski et al. (2012) [63]
		R: Gctgaaacggtgcccttgaaac	
*Cecropin 1*	X70030	F: Gcgggttggctgaagaag	Gomulski et al. (2012) [63]
		R: Cggtggctgcgacattag	
*Attacin A*	FC614	F: Aaagtgtctacctctcgtttctgg	Gomulski et al. (2012) [63]
		R: Gcatagtagccactcaagtatcgc	
*PGRP-LC*	HC731	F: Gcacacaccaaaggctacaatc	Gomulski et al. (2012) [63]
		R: Cacccaaacgaagaccctcatc	
*Hsp70*	Y08955.1	F: Ctgccgcagctttagcttac	http://simgene.com/Primer3
		R: Gtctgaactcctctgccaag	
*Hsp23-alpha*	EU870434.1	F: Gtgcgtagcgacgaacaaa	http://simgene.com/Primer3
		R:Agcagttcaagcccagtga	

### 2.8. Statistical analyses

All data are presented as means ± SE. Relative expression data of genes was subjected to a Multifactorial ANOVA with probiotic (LP/AP), concentration, irradiation, and time points as factors. Pupal weight, emergence, and flight ability were analyzed by a Mutifactorial ANOVA with probiotic (LP/AP), and concentration as factors. The means were analyzed by Fisher’s LSD to discriminate means at the 95% confidence level. The data had previously been checked for normality. STATGRAPHICS 18-X64 software was used to analyze the data with a significance level at α = 0.05.

## 3. Results

### 3.1. Relative quantification of *attacin*, *cecropin*, *PGPR-LC*, *hsp23* and *hsp70* gene expression after exposure of medfly males to irradiation

**Fig 2:**
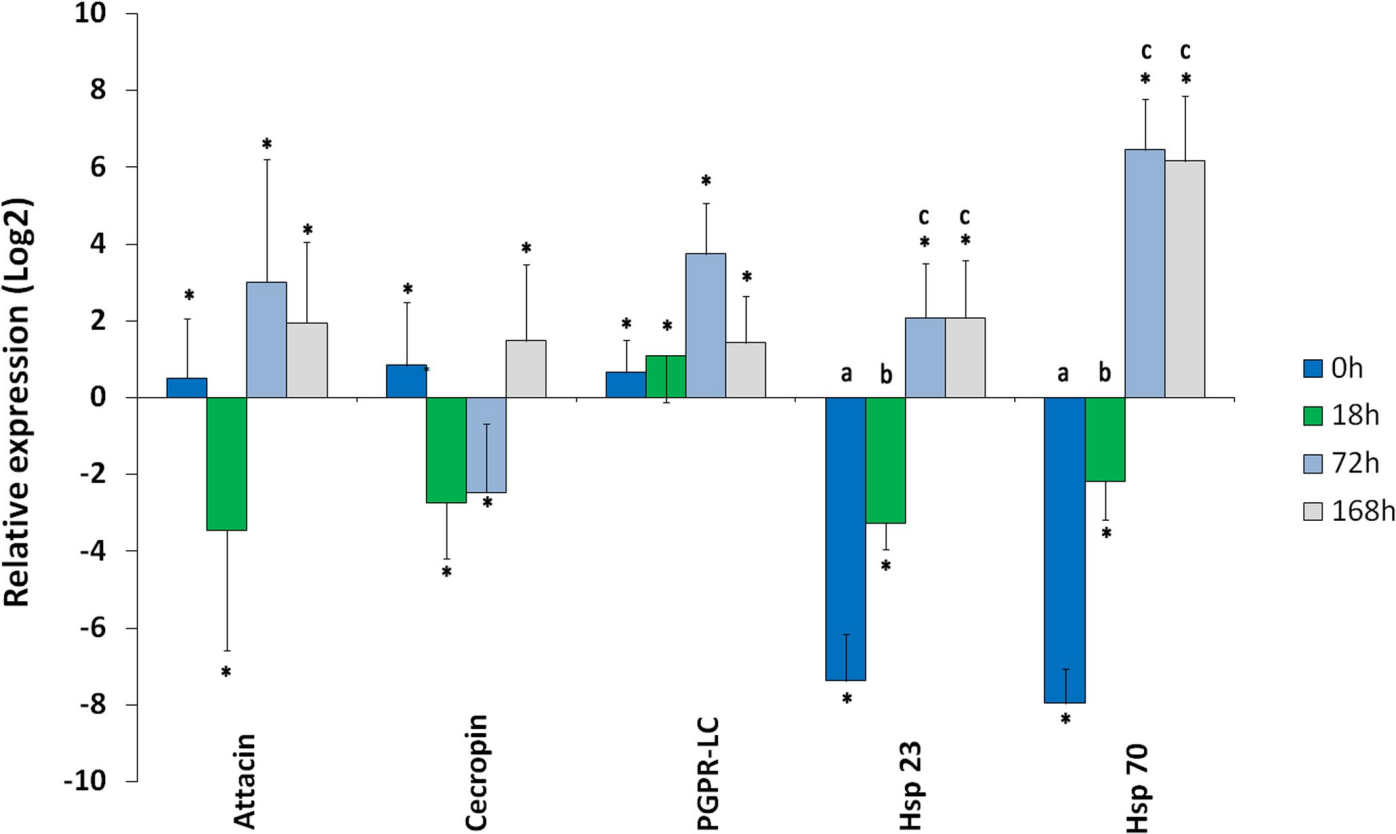
Relative level of expression of *attacin*, *cecropin*, *PGRP-LC*, *hsp23*, and *hsp70* genes determined by qRT-PCR. Barplots showing relative genes expression as means SD of log fold changes from irradiated pupae and sterile adult males aver time compared to the non-irradiated ones. The mRNA level of G6PDH and GAPDH2 transcripts were used to normalize the expression level of candidate genes. Differences in mRNA expression level were analyzed using Multifactor ANOVA followed by LSD to discriminate means at the 95% confidence level. Standard errors across replicate trials are plotted in the graph. Different letters indicate for each gene statistical differences (P≤0.05) observed between means. Symbol (*) above bars indicate for each gene statistical differences (P≤0.05) observed with untreated control.

Induction levels of *attacin*, *cecropin*, *PGPR-LC*,*hsp23* and *hsp70* following medfly irradiation were studied over the time points 0 h, 18 h, 72 h, and 168 h after irradiation (Fig 2). Interaction time x irradiation was significant for *attacin* and for *PGPR-LC*. *Attacin* and *PGRP-LC* showed the same trend with an overexpression at 72 h after irradiation of 3.01 and 3.74-fold, respectively, compared to the control (Fig 2). At 168 h, they both decreased to levels reaching 1.95 and 1.43-fold, respectively. For *cecropin* interaction time x irradiation was significant. *Cecropin* showed a different profile with an important underexpression at 18 h and 72 h (-2.73 and -2.47-fold, compared to the control). At 168 h, it increases to levels of 1.43-fold compared to the control.

For *hsp23* and *hsp70*,interacation time x irradiation was significant. After a significant downregulation due to the irradiation, the two genes start to be upregulated significantly until reaching a maximum level at 72 h (6.45and 2.08-fold; respectively) (Fig 2 and Table 2).

**Table 2:** Statistical analysis of the level of expression of *attacin*, *cecropin*, *PGPR-LC*, *hsp23* and *hsp70* gene, after treatment of medfly males with irradiation by using multifactor ANOVA (P≤0.05).

Gene	Effect	P-values
*Attacin*	Irradiation	**≤0.05**
	Time	0.66
	Time x Irradiation	**≤0.05**
*Cecropin*	Irradiation	**≤0.05**
	Time	0.96
	Time x Irradiation	**≤0.05**
*PGPR-LC*	Irradiation	**≤0.05**
	Time	**≤0.05**
	Time x Irradiation	**≤0.05**
*Hsp23*	Irradiation	**≤0.05**
	Time	**≤0.05**
	Time x Irradiation	**≤0.05**
*Hsp70*	Irradiation	**≤0.05**
	Time	**≤0.05**
	Time x Irradiation	**≤0.05**

### 3.2. Relative quantification of *attacin*, *cecropin*, *PGPR-LC*, *hsp23* and *hsp70* gene expression within probiotic enriched medfly irradiated males

**Fig 3:**
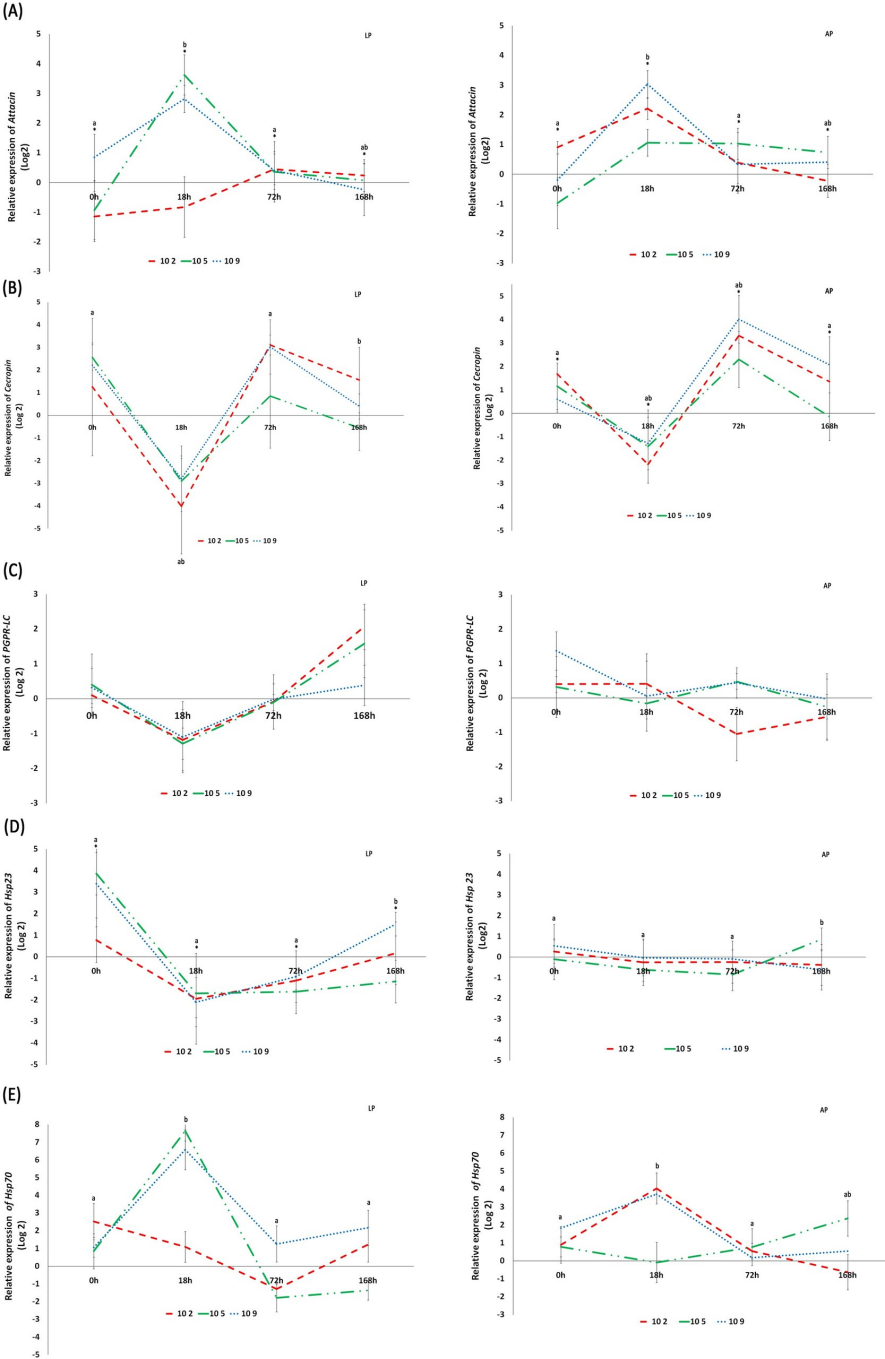
**Relative level of expression of *attacin* (A), *cecropin* (B), *PGRP-LC* (C), *hsp23* (D), *hsp70* (E) genes determined by qRT-PCR.** Line graphs showing relative genes expression as means SD of log fold changes from pupae 0 h, pupae 18 h, adult male 72 h and adult male 168 h age supplemented with live (LP) and autoclaved (AP) probiotic compared to the non-probiotic. The mRNA level of G6PDH and GAPDH2 transcripts were used to normalize the expression level of candidate genes. Differences in mRNA expression level were analyzed using Multifactor ANOVA for each gene followed by LSD to discriminate means at the 95% confidence level. Standard errors across replicate trials are plotted in the graph. Symbol (*) indicates for each gene statistical differences (P≤0.05) observed with untreated control. Different letters indicate statistical difference (P≤0.05) between means.

The relative quantitative real-time PCR-based quantification of the *cecropin*, *attacin*, *PGRP-LC*, *hsp23*, and *hsp70* genes transcripts was analyzed after obtaining males irradiated and enriched with probiotic. The fold induction of the gene transcripts was calculated over four-time points for the sterile enriched live-probiotic (LP) and autoclaved-probiotic adults (AP) at three concentrations compared to the irradiated non-Probiotic ones (Fig 3).

For *attacin*, time and probiotic effects were significant, while concentration was not. However, no significant interaction was observed between these factors. AP and LP individuals were significantly expressed compared to the control. We noticed a significant trend toward overexpression of the transcripts at 18 h that reached for LP adults 3.6 and 2.8-fold (10^5^ and 10^9^, respectively) compared to the control (Table 3 and Fig 3A). The same trend was observed for AP enriched adults.

**Table 3:** Statistical analysis of the level of expression of *attacin*, *cecropin*, *PGPR-LC*, *hsp23* and *hsp70* gene, after treatment of medfly males with probiotic by using multifactor ANOVA (P≤0.05).

Gene	Effect	P-values
*Attacin*	Probiotic LP/AP	**0.0205**
	Time	**0.0000**
	Concentration	0.6
	Probiotic x Time	0.2
	Probiotic x concentration	0.3
	Time x concentration	0.7
*Cecropin*	Probiotic LP/AP	0.09
	Time	**0.0000**
	Concentration	1.3
	Probiotic x Time	**0.03**
	Probiotic x concentration	0.4
	Time x concentration	0.2
*PGPR-LC*	Probiotic LP/AP	0.4
	Time	0.4
	Concentration	0.3
	Probiotic x Time	0.3
	Probiotic x concentration	0.4
	Concentration x Time	0.4
*Hsp23*	Probiotic LP/AP	0.1
	Time	**0.0000**
	Concentration	0.5
	Probiotic x Time	**= 0.05**
	Probiotic x concentration	0.3
	Concentration x Time	0.7
*Hsp70*	Probiotic LP/AP	0.4
	Time	**0.03**
	Concentration	0.9
	Probiotic x Time	**0.0058**
	Probiotic x concentration	0.7
	Concentration x Time	0.9

For *cecropin*, time had a significant effect on the relative expression level, while probiotic and concentration were not significant. On the other hand, the interaction of time x probiotic was significant. There was a remarkable trend of underexpression at 18 h, and overexpression at 72 h after irradiation reaching 3-folds for LP adults (10^2^ and 10^9^, respectively) and 4-folds for AP adults (10^9^), but this trend was not statistically significant (Fig 3B).

On the contrary, for PGRP-LC none of the factors (time, probiotic, and concentration) had an effect on the relative expression level, no significant interactions were observed between the factors. An underexpression of PGPR-LC was observed at 18 h by 1.2-fold compared to the control but only for LP adults (Table 3 and Fig 3C). A trend was then toward an abundance of expression that reached levels up to 1.5-fold at 168 h.

For *hsp23* and *hsp70* genes, the only factor that significantly affected the relative expression level is the time. However, there wasa significant interaction time x probiotic for *hsp23* and for *hsp70* (Table 3).

For *hsp23*, a trend was toward a noticeable decrease of the transcripts at 18 h after an overexpression at 0h for the LP pupae immediately after irradiation that remains non significant (Fig 3D). However, for *hsp70* a modest upregulation of the expression is observed at 0h for both LP and AP pupae (fold varying between 1 to 2.5, and 1 to 1.8; respectively compared to the control). This non-significant trend is significantly accentuated at 18 h reaching an abundance of transcripts of about 7-fold and 4-fold. A decrease is noticed again at 72 h (Fig 3E).

### 3.3. Effect of the probiotic supplementation on quality control parameters of sterile medfly males and females

#### 3.3.1. Pupal weight

**Fig 4:**
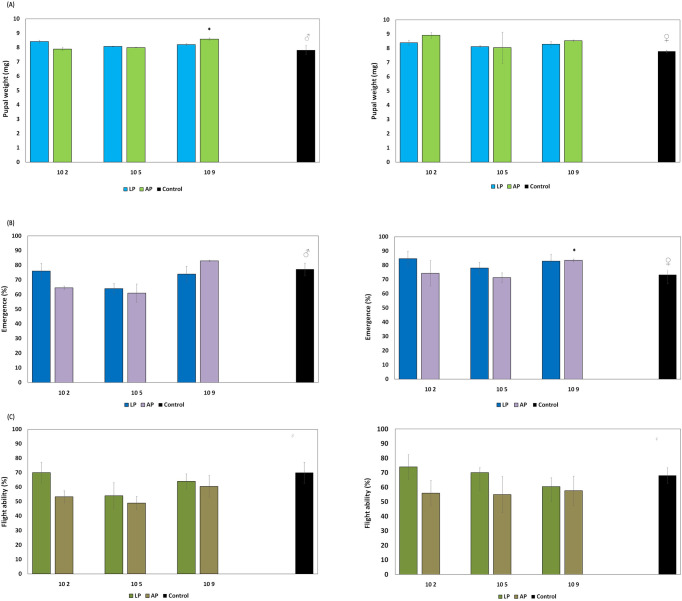
**Pupal weight (A) emergence (B) and flight ability (C) quality control parameters of sterile medfly males and females treated with live (LP) and autoclaved (AP) probiotic at three concentrations 10**^**2**^**, 10**^**5**^** and 10**^**9**^**.** Standard errors across replicate trials are plotted in the graph. Symbol (*) indicates statistical differences (P≤0.05) observed for between groups.

Male pupae weight was improved significantly after the different treatments (F_6,14_ = 7.10, P = 0.007). The highest average weight was recorded for male pupae obtained from the AP enriched diet at10^9^ CFU / ml (8.60±0.28 mg), while the lowest mean pupae weight was recorded for the control (7.83 ± 0.17 mg). On the contrary, the effect was not significant within females (F_6,14_ = 6.14, P = 0.16), with the highest value obtained for AP 10^2^ UFC/ml (89.2±0.19 mg), and the lowest for the control (79.9±5.2 mg) (Fig 4). These obtained averages for sterile males are higher than the acceptable means specified for produced fruit flies for SIT programs (7.5 mg) [60].

#### 3.3.2. Emergence

There was a significant difference between all the treatments (F_6,14_ = 11,34; P = 0,001; F_6,14_ = 3.57, P = 0.01), for both sterile males and females. The highest percentage of emergence recorded in sterile males and females was obtained from the diet enriched with AP bacteria to a concentration of 10^9^ CFU/ml (83.00± 0.56% and 84.59±5.32%). On the other hand, the lowest percentage of emergence for sterile males and for females was recorded in adults obtained from AP bacteria at 10^5^CFU/ml (61±6.1 and 71.33±1.1%). The acceptable mean for sterile male emergence post-irradiation is 70% [60].

#### 3.3.3. Flight ability

All treatments affected significantly the flight ability within sterile males compared to the control (F_6,14_ = 10.33, P≤0.05). LP treatment at 10^2^ and 10^9^ CFU/ml was comparable to the control male flies (70±7.21). However, we noticed a significant reduction in the flight ability for the AP males at 10^2^ and 10^5^ CFU/ml. For females, the highest flight ability percent was obtained with LP adults (10^2^) (73.88±4.92%), compared to the control (68±3.05) (Fig 4), (F_6,14_ = 4.14, P = 0.07). It is worth noting that these values are above the acceptable mean specified for SIT programs being 60% [60].

## 4. Discussion

At the molecular level, immune and stress gene expression has been examined at several time points to determine the effect of irradiation and probiotic enrichmenton medfly larval diet. Biological quality traits, such as pupal weight, emergence, and flight ability were also assessed.

We were able to detect major changes in all of the studied genes expression in medfly males after exposure to irradiation at a dose of 90 Gy. *Attacin* as well as *PGRP-LC* were overexpressed according to our results after exposure to irradiation with a maximum at 72h. Attacinhas been found to be active against Gram-negative bacteria while the role of PGRP family members is the recognition of invading pathogens and the activation and modulation of immune responses [65, 66]. Our results showed that *cecropin* undergoes decreased levels of transcripts within irradiated medfly males at 18 h and 72 h. Cecropins are immune effectors that are synthesized following systemic and local infections [61, 67]. They are known as important agriculturally AMPs that are generated in the fat body and rapidly secreted in the hemolymph of insects [68]. Cecropins are active against Gram-negative and Gram-positive bacteria and fungi [69, 70]. They have long been used as feed additives for poultry and livestock to which they confer immunity against pathogens [71–74].

It is well known that irradiation causes immune response dysregulation for humans and mammals in general [75, 76], while a limited number of studies investigated the effect of irradiation on the immune system, most of them focused on *Drosophila* sp. [56, 77–79]. Moreover, it has been shown that irradiation induced a change in gene expression only for doses that are above 100 Gy [80]. While Zhikrevetskaya et al. [81] reported that after exposition to low doses (5cGy-40cGy), genes could exhibit an overexpression immediately after a 5 cGy irradiation and downregulation after an impact of 40 cGy.

On the other hand, *hsp* transcripts were downregulated after exposure to irradiation at 0 and 18 h and started to increase afterward peaking at 72 h, confirming their role in irradiation-stress tolerance [82]. For medfly, the only data available are those from Anantanawatet al. [83] on the response of the *hsp* genes to heat treatment. A sterile male undergoes a variety of stresses before being released in the field, temperature (at the egg and the larval stage), as well as irradiation must be consideredas important stress factors. The GSS medfly strain is heterozygous for the *tsl* mutation [57]. Anantanawat et al. [83] found that *hsp70* was highly responsive to temperature suggesting them as biomarkers to determine whether the flies experienced heat treatment or not. The same could be investigated for irradiation treatment of medflies for maintaining homeostasis. Moreover, this has been proposed by Shim et al. [82] for the Indian meal moth.

Overall, the irradiation influenced the expression of certain AMPs and *hsps* within male medflies. Although protective, this dysregulation has the potential to significantly reduce fitness and is energetically costly [39, 41].

Probiotics have long been known to execute a biological role in the mammalian gut through their antimicrobial and immunomodulatory properties [84]. Several probiotics and their metabolites were reported to suppress the proliferation of pathogenic bacteria [85, 86], increase the barrier function [87], relieve enteritis [88], and induce thermal-stress tolerance [89]. Within insects they have been mainly used for the feeding of honey bee *Apis mellifera* L. (Hymenoptera: Apidae), with an activation of the humoral immune system to produce AMPs such as *abaecin* and *defensin* [90].

For medfly, *attacin* genes showed a trend toward significant abundance after probiotic enrichment at 18h for live and autoclaved probiotics.In the case of *PGRP-LC*, probiotics had no discernible effect on the expression level. For *cecropin*, we noticed a significant interaction between time and probiotics. The time needed for *cecropin* to be expressed after probiotic enrichment is 18 h after irradiation to reach a maximum at 72 h of 3- and 4-fold for LP and AP 10^9^ bacteria, respectively. Although these findings are significantonly for autoclaved probiotics, they provided evidence that the irradiation reduced the level of *cecropin* transcripts, whereas the probiotics may enable, by a certain mechanism, a slight induction of *cecropin* in the irradiated pupa and young adult stage. Previous studies reported that *cecropin* is secreted an hour after the hoemocoel infection and reaches a maximum after 2–6 h [91], and that its expression is correlated with the bacterial infection [33]. Interestingly, it was reported that low irradiation dose (50 cGy) within *Drosophila* sp. induces the same signaling pathways as infections [56]. In our work, we may have missed an induced expression within a few hours of irradiation in medfly males. Probably, the immune response is expressed rapidly to be detected and differs in function of radiation dose and the time of exposure [81].

In short, we detected a range of responses for this set of genes to irradiation and probiotic enrichment, which could also be attributed to host genetic background as well as stressor agent. The AMP genes may be expressed differently within the same family [92].

We still lack knowledge on the medfly response to the different stressors during the production and irradiation processes, let alone when probiotic is added. However, a similar study on the nematode *Caenorhabditis elegans* fed on killed *Lactobacillus*, noticed overexpression of the *hsp70* gene enhancing tolerance to heat and H_2_O_2_ induced stress [93]. It was also demonstrated according to *in vitro* experiments on humans and rodents that probiotics have a protective action in the gastrointestinal tract through the secretion of hsps. However, these probiotic criteria have been shown to depend on the selected bacteria, *E*. *coli* is capable to induce expressionof gut epithelial hsps, but *Enterobacter aerogenes*, *E*. *faecalis*, and *Proteus mirabilis*, were not [94, 95]. In our case the probiotic strain *Enterobacter* sp. did not induce the secretion of *hsp*23 nor of *hsp*70. This finding supports the use of a consortium of probiotics for the mass rearing of medfly males.

Here we have also shown a positive effect of probiotics on pupal weight within irradiated males, specifically (AP) probiotics. This result confirms the findings obtained by Hamden et al. [28], and Kyristis et al. [96], who added probiotics to medfly larval diet, and Yao et al. [97], who mixed live *Enterobacter* sp. into *Bactrocera cucurbitae* larval diet. On the contrary, Augustinos et al. [29] found that *Enterobacter* sp. didn’t have an effect on medfly pupal weight. The same goes for Khan et al. [98], who added *Proteus* sp. to *Bactrocera dorsalis*. In turn, despite these controversial outcomes, there is a broad consensus in favor of the use of probiotics in insectaries. Furthermore, recent work has shown that the probiotic could substitute the brewer’s yeast [96, 99]. Therenow exists compelling evidence of the use of probiotics for the improvement of some quality traits for mass-reared insects for SIT programs.

The use of probiotics may also be beneficial for medfly RIDL strains (Release of Insects Carrying a Dominant Lethal) [100, 101]. RIDL strains create a female-specific conditional lethality by using the *E*. *coli* tetracycline (Tet) resistance operon to suppress expression (Tet-off) of a lethal effector by feeding the antibiotic during rearing. Antibiotic application in the rearing diet is likely to alter the gut microbiota that could be restored by probiotic supplementation.

Finally, except for pupal weight, where AP gave the highest value, our findings showed no significant difference between live and autoclaved probiotics in inducing gene expression when it happens. Indeed, even inactivated bacteria have been found to interact in the gastrointestinal tract; these fractions are known as parabiotics or autoclaved/ghost probiotics. We could consider these autoclaved probiotics as a parabiotic that was defined by Taverniti and Guglielmetti [102] and later by Ditu et al. [103] *as“non-viable components NVC of microbial origin that exhibit beneficial effects on the health of the human or animal host organism”*. According to Ditu et al. [103] and Adams [104], these fractions are important modulators of the immune response.

## 5. Conclusions

This is a preliminary investigation on the raised immune response when medfly males are exposed to irradiation and probiotic supplementation. We were able to detect a significant effect in expression levels following irradiation within medfly males. The probiotics (live and autoclaved), in turn, influenced the expression of *attacin* only. However a significant interaction between probiotic and time was noticed for the other genes. These results although encouraging, they are far from conclusive about the role of probiotics in changing the immune competence of the host. The definition of a probiotic consortium with complementary actions should be appropriately investigated. Probiotics, whether live or autoclaved, are beneficial for insect rearing. However, using the autoclaved ones (parabiotics) elicit many advantages during mass rearing. They could be easily produced and stored, and they are safer for workers in the facilities and the field.

## Supporting information

S1 Data (XLSX)

S2 Data (XLSX)
